# The complete chloroplast genome of a fern genus *Thelypteris interrupta*

**DOI:** 10.1080/23802359.2019.1710294

**Published:** 2020-01-14

**Authors:** Rahul Vasudeo Ramekar, Ik-Young Choi, Myounghai Kwak, Eun Ju Cheong, Kyong-Cheul Park

**Affiliations:** a Department of Agriculture and Life Industry, Kangwon National University, Chuncheon, South Korea;; b Plant Resources Division, National Institute of Biological Resources, Incheon, South Korea;; c Division of Forest Science, Kangwon National University, Chuncheon, South Korea

**Keywords:** Chloroplast genome, *Thelypteris interrupta*

## Abstract

In this study, we report the complete chloroplast (cp) genome of *Thelypteris interrupta*, a fern member, and comparative analysis with its related family members. The cp genome was 155,983 bp long, with a typical quadripartite structure including a pair of inverted repeat regions (25,614 bp) separated by a large (82,769 bp) and small (21,986 bp) single-copy (SC) region. The genome encodes a total of 88 protein-coding genes, 35 tRNA genes, and 8 rRNA genes. Additionally, we identified 86 RNA editing sites in 52 genes; most of the substitution was U to C (52 sites), while C to U conversion occurred in 34 positions. The phylogenetic analysis strongly supported the relationship of *T. interrupta* with *Ampelopteris prolifera* and *Christella appendiculata* of Thelypteridoideae family.

## Introduction

*Thelypteris interrupta* (Thelypteridaceae), commonly known as *Cyclosorus interruptus*, is a fern species broadly distributed in tropic and sub-tropic regions of all the continents (Sinclair et al. 2012). The leaves of *T. interrupta* are believed to be effective against general sickness, cough, and burns (Quadri-Spinelli et al. 2000). However, in Korea, the natural resources of *T. interrupta* have been overexploited and included in the list of endangered species. Additionally, genomic information of the species is limited, and there is no clear authentication system to distinguish *T. interrupta* from its relatives.

Here, we report the first complete chloroplast (cp) genome sequence of *T. interrupta*. The samples of *T. interrupta* were collected from its natural habitat in Jejudo Island, South Korea. The specimen is stored in the Herbarium of National Institute of Biological Resources, Incheon, South Korea (Voucher number: NIBRVP0000627878). Genomic DNA was extracted following the modified CTAB method (Doyle 1987). After the pair-end library was constructed, whole-genome sequencing was performed using an Illumina HiSeq 4000 platform (Illumina Inc., San Diego, CA, USA). Trimmomatic v0.32 (Bolger et al. 2014) was used to filter and trim reads and Newbler assembler, v2.9 (454 Life Sciences, Branford, CT) for assembling the high-quality reads. The initial annotation of the cp genome was conducted using the DOGMA program (Wyman et al. 2004), and tRNAscan-SE to predict protein-coding genes, transfer RNA genes, and ribosome RNA genes (Lowe and Eddy 1997) We have submitted the assembled and annotated sequence to GenBank under accession number MN599066.

**Figure 1.**
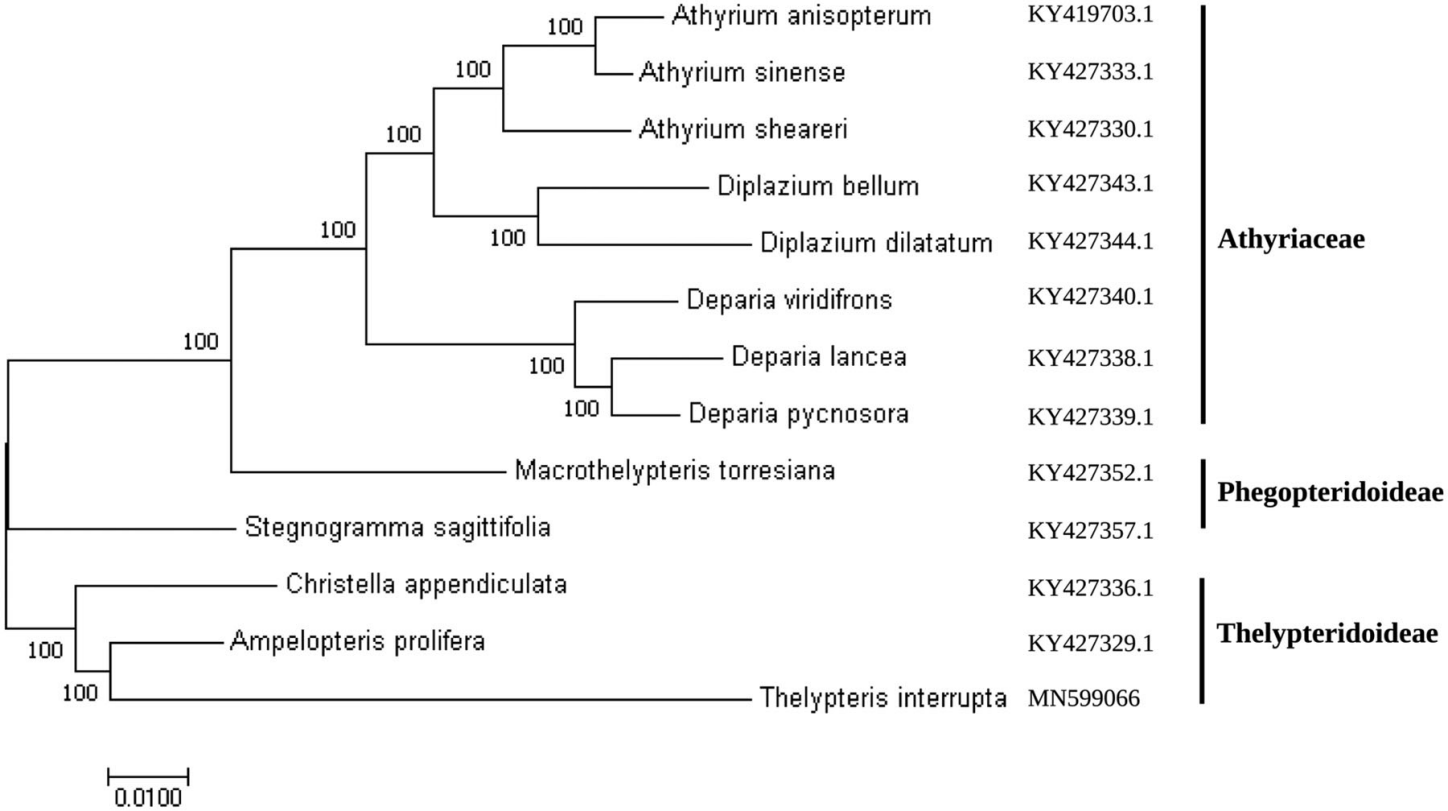
Molecular phylogenetic tree of the fern family Athyriaceae, Phegopteridoideae, and Thelypteridoideae based on the complete cp genome of 13 species.

To investigate the phylogenetic status of *T. interrupta* within the fern family, 12 complete cp genomes belonging to the family Athyriaceae, Phegopteridoideae, and Thelypteridoideae were selected. A neighbor-joining (NJ) tree was constructed with Mega 6.0 using 1000 bootstrap replicates (Tamura et al. 2013). Results clustered the fern species into three groups (Figure 1). All the members of family Athyriaceae (*Athyrium anisopterum*, *Athyrium sinense*, *Athyrium sheareri*, *Diplazium bellum*, *Diplazium dilatatum*, *Deparia lancea*, *Deparia pycnosora*, *Deparia viridifrons*) and one member of Phegopteridoideae family (*Macrothelypteris torresiana*) clustered in one group. *Stegnogramma sagittifolia* (Phegopteridoideae) was placed in distinct clusters while another group comprised members from Thelypteridoideae family (*Christella appendiculata*, *Ampelopteris prolifera*, and *Thelypteris interrupta*). *Thelypteris interrupta*, along with *C. appendiculata* and *A. prolifera* formed a monophyletic clade with a high bootstrap value, indicating a close relationship among these species.

## References

[CIT0001] Bolger AM, Lohse M, Usadel B. 2014. Trimmomatic: a flexible trimmer for Illumina sequence data. Bioinformatics. 30(15):2114–2120. 24695404 10.1093/bioinformatics/btu170 PMC4103590

[CIT0002] Doyle JJ. 1987. A rapid DNA isolation procedure for small amounts of fresh leaf tissue. Phytochem Bull. 19:11–15.

[CIT0003] Lowe TM, Eddy SR. 1997. tRNAscan-SE: a program for improved detection of transfer RNA genes in genomic sequence. Nucleic Acids Res. 25(5):955–964. 9023104 10.1093/nar/25.5.955 PMC146525

[CIT0004] Quadri-Spinelli T, Heilmann J, Rali T, Sticher O. 2000. Bioactive coumarin derivatives from the fern *Cyclosorus interruptus*. Planta Med. 66(8):728–733. 11199130 10.1055/s-2000-9908

[CIT0005] Sinclair S, Stajsic V, Sutter G. 2012. *Cyclosorus interruptus* (Thelypteridaceae): new to Victoria. Muelleria. 30:183–188.

[CIT0006] Tamura K, Stecher G, Peterson D, Filipski A, Kumar S. 2013. MEGA6: Molecular Evolutionary Genetics Analysis version 6.0. Mol Biol Evol. 30(12):2725–2729. 24132122 10.1093/molbev/mst197 PMC3840312

[CIT0007] Wyman SK, Jansen RK, Boore JL. 2004. Automatic annotation of organellar genomes with DOGMA. Bioinformatics. 20(17):3252–3255. 15180927 10.1093/bioinformatics/bth352

